# Dr. Charles O. Chester

**Published:** 1911-01

**Authors:** 


DR. CHARLES O. CHESTER, of Buffalo, died at his home after a prolonged illness, November 22, 1910, aged 54 years. He was born in Buffalo, and graduated from the medical department of the University of Buffalo in 1876. He was surgeon at the Erie County Eye and Ear Infirmary for sixteen years, and until his retirement about two years ago. At the time of his death he was a member of the University of Buffalo Alumni Association. Surviving him is his wife, Ida M. Chester.

